# The High Seropositivity of Mumps Virus IgG Antibodies among School-Aged Children in Rural Areas of the Mbarali District in the Mbeya Region, Tanzania: It Is High Time for Consideration in the National Immunization Program

**DOI:** 10.3390/children11010073

**Published:** 2024-01-09

**Authors:** Mariam M. Mirambo, Fausta Michael, Helmut Nyawale, Frank Mbugano, Maneja B. Walwa, Dina Mahamba, Delfina R. Msanga, Bernard Okamo, Prisca Damiano, Stephen E. Mshana

**Affiliations:** 1 Department of Microbiology and Immunology, Catholic University of Health and Allied Sciences, Mwanza P.O. Box 1464, Tanzania; helmut.nyawale@bugando.ac.tz (H.N.); frank.bugano@bugando.ac.tz (F.M.); maneja.walwa@bugando.ac.tz (M.B.W.); priscadamian@bugando.ac.tz (P.D.); mshana72@bugando.ac.tz (S.E.M.); 2 Ministry of Health, Immunization and Vaccine Development Program, Dodoma P.O. Box 743, Tanzania; fausta.selemani@afya.go.tz; 3 Department of Pediatrics and Child Health, College of Health Sciences, University of Dodoma, Dodoma P.O. Box 395, Tanzania; dinamahamba@udom.ac.tz; 4 Department of Pediatrics and Child Health, Weill Bugando School of Medicine, Catholic University of Health and Allied Sciences, Mwanza P.O. Box 1464, Tanzania; delfina.msanga@bugando.ac.tz; 5 Department of Biochemistry and Molecular Biology, Catholic University of Health and Allied Sciences, Mwanza P.O. Box 1464, Tanzania; bernard_okamo@bugando.ac.tz

**Keywords:** mumps virus, seroprevalence, Mbeya, Tanzania

## Abstract

Mumps is an acute contagious viral disease caused by paramyxovirus characterized by complications that include orchitis, oophoritis, aseptic meningitis, and spontaneous abortion among many others. This study reports high mumps IgG seropositivity among school-aged children in rural areas of the Mbeya region, information that might be useful in understanding the epidemiology of mumps and instituting appropriate control measures including vaccination. Between May and July 2023, a cross-sectional study involving 196 enrolled children aged 5–13 years was conducted. Sociodemographic information and other relevant information were collected using a structured data collection tool. Blood samples were collected and used to detect mumps immunoglobulin G antibodies using indirect enzyme-linked immunosorbent assay (ELISA). A descriptive analysis was performed using STATA version 15. The median age of the enrolled children was 13 (interquartile range (IQR): 8–13) years. The seropositivity of mumps IgG antibodies was 88.8% (174/196, 95% CI: 83.5–92.5). By multivariable logistic regression analysis, history of fever (OR: 5.36, 95% CI: 1.02–28.22, *p* = 0.047) and sharing utensils (OR: 8.05, 95% CI: 1.99–32.65, *p* = 0.003) independently predicted mumps IgG seropositivity. More than three-quarters of school-aged children in rural areas of the Mbeya region are mumps IgG-seropositive, which is significantly associated with the sharing of utensils and history of fever. This suggests that the virus is endemic in this region, which calls for further studies across the country so as to institute evidence-based, appropriate control measures including a vaccination program.

## 1. Introduction

The mumps virus is an enveloped ribonucleic acid (RNA) virus that belongs to the genus rubulavirus from the family paramyxoviridae [1]. The mumps virus is acquired through respiratory droplets, with primary replication happening in the nasopharynx and regional lymph nodes [2]. Viremia happens after 12–25 days and lasts between 3 and 5 days. This is followed by the spread of the virus to multiple tissues, including salivary glands, meninges, testes, pancreas, and ovaries, resulting in the inflammation of these organs.

Despite the inflammation of infected organs/tissues (parotitis, orchitis, meningitis, oophoritis, pancreatitis, mastitis, encephalitis, etc.) and associated complications, the mumps infection can lead to spontaneous abortion if contracted in the first trimester of pregnancy. Other complications of mumps include nephritis, myocarditis, and other sequelae such as cranial nerve palsies, seizures, paralysis, and hydrocephalus [3,4,5]. Orchitis has been reported as the most common complication, ranging from 6% among vaccinated post-pubertal males to 30% among unvaccinated post-pubertal males. Orchitis is usually associated with acute tender testicular swelling, fever, nausea, and vomiting. Testicular atrophy occurs in about half of patients, with a reported theoretical risk of sterility based on the pathogenesis [6].

The mumps virus infection is asymptomatic in the majority of cases and infections are distributed worldwide, with seropositivity ranging from 50% to 90% in children aged 4–15 years old [3,7]. Mumps infection rates in areas without immunization have been reported to range between 100 and 1000 cases per 100,000 people, which is characteristic of many viral infections with epidemic peaks every 2–5 years [8]. Globally, more than 560,000 cases of mumps infections were reported between 2005 and 2010 [9]. Several factors have been documented to contribute to mumps infection. These factors include age; exposure; compromised immunity; time of year; travel; sharing objects containing infected saliva, such as toys, cups, plates, spoons, forks, etc.; and vaccination status [10,11]. Furthermore, immunocompromised children are at an increased risk of transmission; travel to areas where vaccination rates are low can also increase the risk of transmission [12].

The incidence of acquiring the mumps virus infection is higher in countries with no MMR vaccination than in countries with a mumps vaccination program. Seropositivity for IgG antibodies for the mumps virus was found to be 69.4% before MMR vaccination and as high as 92% after vaccination [11,13]. This indicates that immunization status increases protection against mumps virus infections. Overcrowding has been shown to increase the risk of contracting a mumps infection and rural populations are most affected because of the large number of people living in a household of more than five [14,15,16,17].

The live, attenuated mumps vaccine was licensed for use in 1967 with a combination of measles, mumps, rubella, and varicella (MMRV) in 2005. Vaccine implementation has reduced the number of cases, with approximately 94% of those receiving a single dose seroconverted with measurable antibody titers. The real-world data post-licensure have the effectiveness of one dose of the mumps vaccine to be 78% and that of two doses to be 88%. By 2017, 121 countries, including the majority of America and Europe, had introduced the mumps vaccine, with 73 countries not yet having introduced it, and the majority of these countries were in Africa and Asia [18]. African countries that have implemented the mumps vaccination include Algeria, Seychelles, Mauritius, and Carbo Verde [19,20].

Vaccine implementation requires epidemiological data to understand the magnitude of the disease and its associated complications. The WHO recommends routine mumps vaccination in countries with >80% coverage of measles and rubella vaccination, for which Tanzania has achieved >90% coverage (https://immunizationdata.who.int/pages/profiles/tza.html, accessed on 15 December 2023). The absence of vaccine implementation in Tanzania and the scarcity of epidemiological data necessitate the need for more studies to understand the magnitude of the disease. To date, only two studies have documented the prevalence of the mumps virus in Tanzania [21,22].

Understanding the epidemiology of infection is one of the key steps in devising control measures. The current study provides information on the magnitude of mumps IgG antibodies using EIA and their associated factors among school-aged children from the Mbeya region in Tanzania, information that might be useful in future prevention strategies.

## 2. Materials and Methods

### 2.1. Study Design, Duration, Study Area, and Study Population

A cross-sectional study involving school children aged 5–13 years was conducted from May to July 2023 in selected public primary schools in rural areas of the Mbarali district in the Mbeya region. The schools included Ubaruku Primary School, Utyego Primary School, Mbarali Primary School, and Mkobwe Primary School. The total number of children in each school was 1480, 1159, 921, and 950 for Ubaruku Primary School, Utyego Primary School, Mbarali Primary School, and Mkobwe Primary School, respectively.

### 2.2. Sample Size Estimation, Sampling Procedures, and Selection Criteria

The Kish Leslie formula was used to calculate the sample size, using a seropositivity of 77% from a previous study conducted in Dar es Salaam, Tanzania, among school-aged children [22], with a tolerable error of 7%. The minimum sample size was 139 children; however, we enrolled a total of 196 school-aged children using a stratified multistage cluster sampling technique. All children between the ages of 5 and 13 years whose parents agreed to participate in the study were enrolled, while those who failed to provide blood samples were excluded from the study.

### 2.3. Data Collection, Sample Collection, and Laboratory Procedures

A pretested, structured data collection tool was used to collect child information, which included sociodemographic information (e.g., age, sex, residency, parent’s/guardian’s education, etc.) and other relevant information such as comorbidities, body temperature, and vaccination status. Under aseptic procedures, 3–5 mL of venous blood was drawn from each participant and placed in well-labeled, plain vacutainer tubes (Becton Dickson Ltd., Franklin Lakes, NJ, USA). Samples were collected and placed in a cool box at 4–8 °C for temporary storage, then transported to Mbarali District Hospital where sera were extracted and stored in cryovials at −30 °C. Samples were triple-packed as per standard operating procedures and transported from Mbeya to the Catholic University of Health and Allied Sciences (CUHAS) microbiology laboratory.

### 2.4. Laboratory Procedures

The detection of mumps IgG antibodies was performed using an indirect enzyme-linked immunosorbent assay [23] as per the manufacturer’s instructions (Vircell, S.L. parque Tecnologico de la Salud, Avicena, Granada, Spain). The assay has a sensitivity of 95% and a specificity of 95%. Briefly, water bath was set to 37 °C and all reagents were brought to room temperature before use. A total of 100 μL of serum diluent was added to all wells, followed by the addition of 5 μL of each sample, 5 μL of the positive control, 5 μL of the cut-off control (in duplicate), and 5 μL of the negative control into the corresponding wells. The plate was shaken in a plate shaker for 2 min until a homogenous mixture of the reagents was achieved. The plate was covered with a sealing sheet and incubated at 37 °C for 45 min; then, the seal was removed, and the liquid was aspirated from all wells and washed 5 times with 0.3 mL of washing solution per well as per manufacturer’s instructions. Immediately, 100 μL of IgG conjugate solution was added into each well; plates were covered with a sealing sheet and incubated at 37 °C for 30 min. The seal was removed, and the liquid was aspirated from the wells and washed with 0.3 mL of washing solution per well, and any remaining liquid was drained off. Immediately, 100 μL of substrate solution was added to each well and incubated at room temperature for 20 min, followed by the addition of 50 μL of stopping solution to all wells. The plate was read at 450/620 nm with a spectrophotometer within 1 h of stopping.

### 2.5. Data Management and Analysis

Laboratory results were recorded in the ELISA logbook and then transferred to a Microsoft Excel sheet for cleaning and coding. Data analysis was performed using STATA version 15 (College Station, TX, USA: StataCorp LLC). Percentages or fractions were used to summarize categorical variables, while the mean with standard deviation and median with interquartile range were used for continuous variables where appropriate. Prevalence was calculated by dividing the number of confirmed mumps IgG-seropositive samples by the total number of tested samples, with the 95% confidence interval calculated using a two-sample test of proportions. Univariable and multivariable analyses using logistic regression were conducted to determine factors associated with the presence of mumps antibodies, whereby factors with a *p*-value of <0.2 on univariable analysis were subjected to multivariable analysis. Odd ratios and 95% confidence intervals were determined and the variables with a *p*-value of <0.05 were considered statistically significant.

## 3. Results

### 3.1. Sociodemographic, Clinical, and Other Characteristics

A total of 196 children aged 5–13 years were enrolled from four different primary schools; 48% (99/196) were enrolled from Mbarali Primary School. The median age of the enrolled children was 13 (IOR: 8–13) years. More than half (63.3%, 124/196) of the children were female. Regarding the education levels of the parents, 47.9% (94/196) of mothers had primary education while more than half (57.7%, 113/196) of fathers had secondary education. More than three-quarters (79.1%, 155/196) of fathers of enrolled children were farmers. The median number of siblings of the participants who were enrolled in this study was 4 (IQR: 2–8) (Table 1).

Most of the school-aged children that were enrolled (82.7%, 162/196) had a normal body mass index (BMI) and more than one-third of study participants (44.4%, 87/196) reported a history of mumps. More than one-third of participants (36.7%, 72/196) reported no clinical signs suggestive of parotitis, while about 47.5% (93/196) had a history of fever. Moreover, only 2.5% (5/196) had muscle aches and 3.6% (7/196) had a sore throat. More than two-thirds (72.4%, 163/196) of the children reported having a habit of sharing utensils (Table 2).

### 3.2. The Seropositivity of Mumps IgG Antibodies and Associated Factors among School-Aged Children in Mbeya, Tanzania (N = 196)

The seropositivity of mumps IgG antibodies was 88.8% (174/196, 95% CI: 83.5–92.5). In the univariable logistic regression analysis, an increased number of siblings (OR: 1.89, 95% CI: 1.21–2.95, *p* = 0.005), the mother’s education (OR: 14.5, 95% CI: 3.82–55.09), the mother’s employment status (OR: 0.12, 95% CI: 0.03–0.49, *p* = 0.003), history of fever (OR: 10.96, 95% CI: 2.49–48.34, *p* = 0.002), history of parotitis (OR: 5.62, 95% CI: 2.09–15.13, *p* = 0.001), and sharing utensils (10.98, 95% CI: 4.00–13.16, *p* < 0.001) were significantly associated with mumps IgG seropositivity. In the multivariable logistic regression analysis, history of fever (OR: 5.36, 1.02–28.22, *p* = 0.044) and sharing utensils (OR: 8.05, 95% CI: 1.99–32.65, *p* = 0.003) independently predicted mumps IgG seropositivity among school-aged children in Mbeya, Tanzania (Table 3).

## 4. Discussion

The availability of seroepidemiological data is the key to understanding levels of natural immunity, which is important in devising control strategies. Tanzania is among the countries where mumps vaccination is not implemented and has limited epidemiological data. This study documents a high seropositivity of mumps IgG antibodies among school-aged children in rural areas of the Mbarali district in the Mbeya region, Tanzania.

In this study, more than three-quarters (88.8%) of the children were found to be mumps IgG-seropositive, indicating prior exposure to the mumps virus. The seropositivity reported in this study is comparable to a previous report in the city of Dar es Salaam, which reported a seropositivity of 77% [22]. This similarity might be due to the enrollment of children with the same age range since mumps seropositivity tends to increase with age [3]. In addition, the two studies used the same assays, making the data more comparable. In comparison to previous reports elsewhere, the seropositivity reported in the current study is also comparable to 91.6% in Colombia, 89.1% in Yemen, 80.2% in Iran, 71.9% in Turkey, and 69.4% in Brazil [13,24,25,26,27,28,29]. However, in comparison to a previous report in Mwanza that reported a seropositivity of 21.4%, the seropositivity reported in the current study is indeed high [21]. Possible explanations for the variations could be differences in study populations, geographical and climatic conditions, and seasonality. The current study was conducted in the Mbarali district from May to July, which is a dry and cold seasonal time. It has been previously documented that mumps seropositivity tends to be very high during winter in temperate regions, while, in hot climates, transmission can be high at any time of the year [10,21]. Further studies to establish the relationship between mumps seropositivity and seasonality are warranted in Tanzania across different regions. Given the fact that mumps vaccination is not implemented in Tanzania, the high seropositivity observed in the current study indicates that the virus is endemic and might contribute to the prevalence of related complications. Further studies to establish a causal effect relationship are warranted in Tanzania. Moreover, the current study confirms that the mumps virus is common in rural areas in Tanzania compared to urban settings, which was also observed in our previous report in the Mwanza region [21]. A possible explanation for this could be the fact that most viral infections including the mumps virus are associated with low living standards, which is most common in rural areas in Tanzania and other low-income countries. Moreover, most of the parents of these children had low education levels and most of them were farmers, signifying low socioeconomic status, as previously reported [27]. Based on these findings, it is high time for low- and middle-income countries including Tanzania to consider including the mumps vaccine in the Expanded Program for Immunization (EPI).

Among the factors studied, the sharing of utensils was found to predict mumps IgG seropositivity among school-aged children in Mbeya. This observation corroborates a previous report in Mwanza [21]. A possible explanation for this could be that mumps is a highly contagious viral infection that spreads through respiratory droplets, saliva, and direct contact with contaminated objects. The mumps virus can persist on inanimate objects for hours to days [30]. As previously documented, sharing utensils, especially when they come into contact with an infected person’s saliva or respiratory secretions, can facilitate the transmission of the mumps virus [1,31].

In the current study, a history of fever predicted mumps IgG seropositivity, which is inconsistent with previous reports by Rakiru et al. in Mwanza and Nkinda et al. in Dar es Salaam among unvaccinated children [21,22]. Moreover, the current study did not find any association between previous history of parotid gland enlargement and IgG seropositivity. As previously reported, mumps occurs asymptomatically in 20–30% of cases and some sufferers may have non-specific symptoms [32].

The limitations of this study include recall bias due to the fact that a long time elapsed between the event and the enrollment time; this could explain the lack of an association of many clinical factors with mumps IgG seropositivity.

## 5. Conclusions

More than three-quarters of school-aged children in rural areas of the Mbarali district in the Mbeya region were found to have been previously exposed to the mumps virus, and this exposure was significantly associated with the sharing of utensils and a previous history of fever. Further multi-center studies to understand the epidemiology of the mumps virus in Tanzania and associated complications are warranted. Such studies will justify the need to introduce the MMR vaccine into the national immunization program in order to prevent mumps virus infections and reduce associated complications. This is important due to the fact that in case of any pandemics like COVID-19, outbreaks of diseases might occur, as evidenced by large mumps virus outbreaks in Pakistan [33]. The country needs to consider the Strategic Advisory Group of Experts on Immunization (SAGE) recommendations that require (i) a careful review of the uncertainties, risks, and programmatic implications when considering the introduction of mumps-containing vaccines in LMICs, (ii) the introduction of the mumps vaccine in combination with measles and rubella as an MMR vaccine delivered using the same schedule as for MR vaccines and maintaining a high coverage as recommended for MR vaccines to mitigate the risk of a shift in age-specific incidences of infection, and (iii) enhanced surveillance of mumps infections and related complications to demonstrate the impact of the vaccine and monitor epidemiological shifts (https://cdn.who.int/media/docs/default-source/immunization/sage/2023/september/sage_sept2023_meeting_highlights.pdf?sfvrsn=5ac08c01_4, accessed on 15 December 2023). Furthermore, more studies to establish the transmission of mumps virus and genotypes across Tanzania and Africa, as recently reported in Europe [34], are needed to provide extensive data regarding the circulating genotypes. These data are necessary to monitor the impact of vaccination programs and the evolution of the virus towards vaccine-escaping mumps virus strains [35].

## Figures and Tables

**Table 1:** Sociodemographic characteristics of the enrolled participants in Mbeya (*N* = 196).

Sociodemographics Data			
Variables/Characteristics		Frequency (n)	Percent (%)
Median [IQR] Age (yrs)		13 (8–13)	
Median [IQR] Number of Siblings		4 (2–8)	
Sex	Female	124	63.3
	Male	72	36.7
School	Mbarali	96	48.9
	Mkobwe	33	16.8
	Ubaruku	19	9.7
	Utyego	48	24.5
Father’s Education	None	13	6.6
	Primary	60	30.6
	Secondary	113	57.7
	Tertiary	10	5.1
Mother’s Education	None	23	11.7
	Primary	94	47.9
	Secondary	66	33.7
	Tertiary	13	6.6
Father’s Occupation	Farmer	155	79.1
	Business	17	8.7
	Employed	12	6.1
	Others	12	6.1
Mother’s Occupation	Farmer	56	28.6
	Business	30	15.3
	Employed	13	6.6
	Housewife	42	21.4
	Tailor	55	28.1

**Table 2:** Clinical characteristics of enrolled children in Mbeya (*N* = 196).

Variable/Characteristics		Frequency (n)	Percent (%)
BMI Status	Normal (18–21 kg/m^2^)	162	82.7
	Underweight (13–<18 kg/m^2^)	33	16.8
	Overweight (>21 kg/m^2^)	1	0.51
History of Mumps Vaccination	Yes	0	0
	No	196	100
History of Having Mumps Disease	No	109	55.6
	Yes	87	44.4
Palor	No	193	98.5
	Yes	3	1.5
Fatigue	No	180	91.8
	Yes	16	8.2
Headache	No	175	89.3
	Yes	21	10.7
Loss of Appetite	No	184	93.9
	Yes	12	6.1
Muscle Ache	No	191	97.5
	Yes	5	2.5
Fever	No	103	52.5
	Yes	93	47.5
Puffy Face	No	148	75.5
	Yes	48	24.5
Parotitis	No	188	95.9
	Yes	8	4.1
Previous Parotitis	No	72	36.7
	Yes	124	63.3
Painful Swallowing	No	193	98.5
	Yes	3	1.50
Sore Throat	No	189	96.4
	Yes	7	3.6
Sharing Utensils	No	50	25.5
	Yes	146	74.5

**Table 3:** Univariable and multivariable logistic regression analyses of factors associated with mumps IgG seropositivity among school-aged children in Mbeya (*N* = 196).

Variable/Characteristics		Mumps Results		Logistic Regression			
		Negative	Positive	Univariable		Multivariable	
		Frequency (%)	Frequency (%)	OR [95% CI]	*p*-Value	OR [95%CI]	*p*-Value
Median [IQR] Age (yrs)			13 (8–13)	0.94 [0.58–1.55]	0.822	0.66 [0.34–1.26]	0.207
Median [IQR] Class			7 (3–7)	1.01 [0.57–1.78]	0.969		
Median [IQR] Number of Siblings			4 (2–8)	1.89 [1.21–2.95]	0.005	1.62 [0.92–2.85]	0.096
Median [IQR] BMI (kg/m^2^)			20.5 (14.3–25.1)	0.97 [0.77–1.22]	0.775		
Sex	Female (124)	11(8.90)	113 (91.10)	1			
	Male (72)	11 (15.30)	61 (84.70)	0.54 [0.22–1.32]	0.175	0.28 [0.07–1.09]	0.066
BMI Status	Normal (162)	17 (10.50)	145 (89.50)	1			
	Underweight (33)	5 (15.20)	28 (84.80)	0.66 [0.22–1.93]	0.443		
	Overweight (1)	0 (0.00)	1 (100.00)	–			
School	Mbarali (96)	8 (8.30)	88 (91.70)	1			
	Mkobwe (33)	2 (6.10)	31 (93.90)	1.41 [0.28–7.00]	0.675		
	Ubaruku (19)	0 (0.00)	19 (100.00)	–			
	Utyego (44)	12 (27.30)	32 (72.70)	0.27 [0.10–0.72]	0.009		
Residence	Mbarali (54)	8 (14.80)	46 (85.20)	1			
	Mkobwe (33)	2 (6.10)	31 (93.90)	2.70 [0.54–13.55]	0.229		
	Ubaruku (20)	0 (0.00)	20 (100.00)	–			
	Utyego (48)	12 (25.00)	36 (75.00)	0.52 [0.19–1.41]	0.200		
	Mpakani (41)	0 (0.00)	41 (100.00)	–			
Mother’s Education	Tertiary (1)	7 (53.80)	6 (46.20)	1			
	None (23)	1 (4.30)	22 (95.70)	25.67 [2.62–251.30]	0.005		
	Primary (94)	7 (7.40)	87 (92.60)	14.5 [3.82–55.09]	0.000		
	Secondary (66)	7 (10.60)	59 (89.40)	9.83 [2.57–37.66]	0.001	1.64 [0.93–2.92]	0.090
Mother’s Occupation	Farmer (55)	5 (9.10)	50 (90.90)	1			
	Business (33)	5 (16.70)	25 (83.30)	0.50 [0.13–1.89]	0.307		
	Employed (10)	6 (46.20)	7 (53.80)	0.12 [0.03–0.49]	0.003		
	Housewife (42)	3 (7.10)	39 (92.90)	1.30 [0.29–5.78]	0.730		
	Tailor (56)	3 (5.40)	53 (94.60)	1.77 [0.40–7.78]	0.452	1.26 [0.95–1.68]	0.113
Muscle Ache	No (191)	21 (11.00)	170 (89.00)	1			
	Yes (5)	1 (20.00)	4 (80.00)	0.49 [0.05–4.63]	0.537		
Fever	No (103)	20 (19.40)	83 (80.60)	1			
	Yes (93)	2 (2.20)	91 (97.80)	10.96 [2.49–48.34]	0.002	5.36 [1.02–28.22]	0.044
Previous Parotitis	No (72)	16 (22.20)	56 (77.80)	1			
	Yes (124)	6 (4.80)	118 (95.20)	5.62 [2.09–15.13]	0.001	1.16 [0.28–4.47]	0.835
Sore Throat	No (189)	21 (11.10)	168 (88.90)	1			
	Yes (7)	1 (14.30)	6 (85.70)	0.75 [0.09–6.54]	0.795		
Sharing Utensils	No (50)	16 (32.00)	34 (68.00)	1			
	Yes (146)	6 (4.10)	140 (95.90)	10.98 [4.00–31.6]	0.000	8.05 [1.99–32.65]	0.003

## Data Availability

The data presented in this study are available in article.
